# Individuals’ explanations for their persistent or recurrent low back pain: a cross-sectional survey

**DOI:** 10.1186/s12891-017-1831-7

**Published:** 2017-11-17

**Authors:** Jenny Setchell, Nathalia Costa, Manuela Ferreira, Joanna Makovey, Mandy Nielsen, Paul W. Hodges

**Affiliations:** 1 School of Health and Rehabilitation Sciences, St Lucia, QLD 4072 Australia; 2 Institute of Bone and Joint Research/The Kolling Institute, Sydney Medical School, The University of Sydney, St Leonards, NSW 2065 Australia

**Keywords:** Pain trajectories, Discourse analysis, Lumbar, Patient perspectives, Psychosocial

## Abstract

**Background:**

Most people experience low back pain (LBP), and it is often ongoing or recurrent. Contemporary research knowledge indicates individual’s pain beliefs have a strong effect on their pain experience and management. This study’s primary aim was to determine the discourses (patterns of thinking) underlying people’s beliefs about what causes their LBP to persist. The secondary aim was to investigate what they believed was the source of this thinking.

**Methods:**

We used a primarily qualitative survey design: 130 participants answered questions about what caused their LBP to persist, and where they learned about these causes. We analysed responses about what caused their LBP using discourse analysis (primary aim), and mixed methods involving content analysis and descriptive statistics to analyse responses indicating where participants learnt these beliefs (secondary aim).

**Results:**

We found that individuals discussed persistent LBP as 1) due to the body being like a ‘broken machine’, 2) permanent/immutable, 3) complex, and 4) very negative. Most participants indicated that they learnt these beliefs from health professionals (116, 89%).

**Conclusions:**

We concluded that despite continuing attempts to shift pain beliefs to more complex biopsychosocial factors, most people with LBP adhere to the traditional biomedical perspective of anatomical/biomechanical causes. Relatedly, they often see their condition as very negative. Contrary to current “best practice” guidelines for LBP management, a potential consequence of such beliefs is an avoidance of physical activities, which is likely to result in increased morbidity. That health professionals may be the most pervasive source of this thinking is a cause for concern. A small number of people attributed non-physical, unknown or complex causes to their persistent LBP – indicating that other options are possible.

## Background

Low back pain (LBP) is the leading musculoskeletal problems contributing significantly to personal and community health burden [1]. Around 40% of people globally experience LBP. For many it is persistent, recurrent and bothersome [2–4]. Reducing the impact of ongoing LBP is a major research priority – with research of trajectories over time an underexplored aspect [5]. Over the last two decades, there has been a comprehensive shift in the understanding of why LBP becomes persistent or recurrent [6, 7]. A large body of research has taken understandings of persistent pain from the predominantly biomedical model that argues LBP is reducible to anatomical or biomechanical factors, to a biopsychosocial model where persistent pain is considered to be largely modified/maintained by a complex combination of biological, psychological and social factors [8–11] that interact. For example, moderation of the biological processes of sensitisation by psychosocial features [10, 12–14], or the development of ongoing musculoskeletal conditions due to avoidance of activity resulting from a fear of exacerbating pain/damage (e.g. the Fear-Avoidance Model outlined by Lethem, Slade, Troup and Bentley [15]).

The clear linking of psychosocial factors with persistent pain highlights that how people think about their pain is an important predictor of severity and chronicity [16, 17]. Amongst other factors, pain beliefs contribute to prognosis [18]. For example, in a study on 1591 patients attending general practices, poor clinical outcomes 6 months after initial consultation were more likely in individuals who expected their LBP to last a long time, perceived serious consequences, and believed they had little control over their pain [19]. Further, excessively negative orientation towards pain correlates with greater pain [20, 21]. People’s beliefs about how physical activity and work affect their LBP account for variance in pain and disability [22–24]. Recent best practice guidelines reflect that understanding and addressing these pain beliefs is an important component of reducing the burden of LBP (e.g., [25]).

This psychosocial understanding of painful conditions, including LBP, is inconsistently reflected in the beliefs and practices of clinicians or people with LBP. Parsons, Harding, Breen, Foster, Tamar, Vogel and Underwood [26] reviewed 22 qualitative studies concluding that physician’s beliefs about painful musculoskeletal conditions were primarily biomedical and, in a cross-sectional study of 453 musculoskeletal physiotherapists, Bishop and Foster [27] highlighted that while these clinicians often recognised the importance of psychosocial factors they contradicted evidence based best practice guidelines by frequently highlighting biomedical over psychosocial factors of LBP cases. Observational studies show clinicians and patients consider risk factors for a sudden onset of LBP to be mainly biomechanical and rarely endorse psychosocial risk factors [28, 29]. Little research has considered why these beliefs and practices persist regarding LBP despite evidence this view is outdated and inaccurate.

There is insufficient knowledge about why people (including clinicians and individuals with the condition) adopt or resist considering pain beliefs (and other psychosocial factors) as contributors to ongoing LBP. One possibility is that the biopsychosocial approach involves a paradigm shift, which challenges (amongst other things) existing biomedical structures and beliefs. Change would necessarily need to be reflected in people’s underlying systems of belief. Little research has investigated these belief systems. The primary aim of this study was to investigate what patterns of thinking (discourses) underlie what people say caused their LBP to persist or recur. The secondary aim was to investigate where people with the condition considered these patterns of thinking came from.

## Methods

### Study design

We created an online survey to elicit individuals with LBP’s understanding of the patterns of their condition including questions about pain increase, condition flare, and persistence or recurrence. We report here on the answers to questions related to individual’s perceptions of why their condition is persistent or recurrent.

### Participant selection

A wide range of participants were purposively sampled via a range of recruitment methods including promotion through pain consumer support organisations, advertisements in local community and health centres and through social media. Inclusion criteria were 1) personal experience of LBP, 2) English language proficiency, and 3) age 18 and over. Efforts were made to include a broad range of participants with LBP by promotion through a variety of sources. Recruitment continued until a satisfactory level of participant diversity and theoretical saturation was achieved. There was no exclusion based on chronicity, currency of LBP, or comorbidities. A total of 130 participants entered full responses to the survey. Most participants were female (74.6%), from Australia (98.5%) and reported daily pain (82.0%). See Table 1 for demographic and LBP details.

**Table 1:** Demographic characteristics of study participants

*Age* (years)	
Mean ± SD	43.2 ± 12.05
*Gender*	
Female	74.6%
Male	25.4%
*Country*	
Australia	98.5%
Other	1.5%
*State*	
Queensland	56.9%
New South Wales	16.9%
Victoria	15.4%
Other	10.8%
*LBP everyday*	
Yes	82%
No	18%
*Time frame of LBP variation*	
Daily	55.4%
Weekly	23.1%
Monthly	7.7%
Other	13.8%
*Periods of no LBP*	
Yes	29.7%
No	70.3%

### Data collection/procedure

The study gained institutional ethics approval. Data were collected through an online survey, which participants consented to enter after reading an information page. They responded to two questions designed to address the aims of this study:

1) What is your understanding of why your low back pain is persisting or recurring? This question required a text-box response with no word limit.

2) Where does this understanding come from? Participant could choose one or more of five options that were provided for this question i) health care provider, ii) internet, iii) family, iv) friends, or v) other. In the final option, ‘other’, short text-box responses were allowed.

### Methodology and theoretical underpinnings

The primary methodology in this study is discourse analysis [30]. This qualitative methodology offers an approach to investigating people’s patterns of thinking, or systems of belief, that underlie their perceived reasons for LBP persisting or recurring. Discourse analysis is based on the premise that the language we use has a role in creating or constituting reality, rather than simply reflecting it – thus discourses are seen as having real world effects [31]. For example, if someone believe that their LBP is due to tissue damage they may behave in particular ways, such as avoiding certain movements or taking time off work – which will have (greater or lesser) effects on their life. We do not mean to suggest that by identifying discourses we can know what individuals will do based on what they say but rather that certain ways of thinking are likely to produce certain realities more broadly (social constructionism [32]). Thus, discourse analysis does not try to simply summarise individual experiences, or try to find out what ‘really happened’ (in this case what caused their LBP to persist or recur), but rather tries to understand how particular patterns of talking and thinking make certain realities (in this case a particular pain belief and its consequences) more likely [32]. In this research, we sought to understand the particular discourses evident in what participants said make their condition persist or recur. This type of analysis has been used frequently in healthcare and offers different insights compared to other forms of analysis [33]. As it has been rarely used to understand painful conditions such as LBP, it has the potential to bring a novel perspective. To consider the second question we used a simple convergent parallel mixed methods design [34], employing a descriptive count analysis (content analysis where data were qualitative, and descriptive statistics were data were quantitative). This analysis produced a descriptive overview of where participants said they learnt about their condition, which we understood from a relativist perspective [32] to be likely (but not certain) to indicate what had occurred in reality.

### Data analysis

Discourse analysis was conducted following procedures outlined by Willig [31]. The six-member analysis team included experienced qualitative and quantitative researchers from a range of disciplines (physiotherapy, medical, psychology, and social work). The analysis was inductive, which means that the research team did not impose a pre-existing theory on the analysis; rather it was the data that drove the development of the discourses [32], although some a priori assumptions are inevitable in any research [32]. The first author, a physiotherapist with social science training and experience in this type of analysis (JS) initially reviewed the entire data set. On a second reading, she formulated four provisional discourses. JS and another author (NC) then independently read the entire dataset and determined which discourses were represented in each participant response. These researchers then discussed to agreement any discrepancies in naming/defining the discourses and coding of the data according to discourse. The other authors then reviewed the entire dataset, and the coding and analysis of findings, with any incongruences discussed to agreement.

We used descriptive statistics to analyse the data from the second question where data were quantitative (sections 1–4), and content analysis where data were qualitative (section 5). The content analysis was initially carried out by JS and then reviewed by the entire team – results were combined using convergent analysis into final groupings so they could be related. We used the Consolidated Criteria for Reporting Qualitative Research (COREQ: [35]) to guide rigour in study design and reporting. Relevant criteria were satisfied for study design and reporting. To improve trustworthiness, data were independently analysed by two team members with other team members providing input into analysis. A researcher external to the study further confirmed trustworthiness and that the results were grounded in the data.

## Results

We report results in three sections: analysis of participant responses to the first and second questions and then the cross analysis of the two.

### Analysis of responses to question 1

We identified four clear patterns of thinking (discourses) in participant’s responses to the question: “What is your understanding of why your low back pain is persisting or recurring?” (Table 2). We describe these discourses below using examples from the data and then highlight their relevance to our research aims in the discussion. Participants are anonymised and differentiated by numbers. Responses were typically one sentence to a paragraph in length. A small number of responses were very short (e.g., one word). All of these were easily identifiable within at least one of the discourses except for two responses “good” P110 and “my employment aggravates it” P16. These two responses were not included in the analysis as their meaning in relation to the four discourses was unclear.

**Table 2:** Discourses found in analysis of participant responses to the question: “What is your understanding of why your low back pain (LBP) is persistent or recurring?”

Discourse (pattern of thinking)	Explanation
1) Body as machine	The body is viewed as biomechanical (literally: the body as a machine) or anatomical. Like a machine, the body is considered to be able to break and can sometimes be repaired. LBP persists because something is physically defective.
2) LBP as permanent/immutable	Related to the first discourse, LBP is conceptualised as a static or fixed entity that once ‘broken’, it cannot be ‘fixed’. LBP is not dynamic or fluid but unchangeable and permanent.
3) LBP is complex	This is a counter discourse to the first two. Multiple factors can contribute to the persistence of LBP – not only biomechanical or anatomical but also possibly psychosocial or cultural factors. There is no simple explanation for ongoing LBP.
4) LBP is very negative	LBP is conceptualised as abnormal, catastrophic, or very negative experience. LBP should be avoided and/or has a large effect on life.

#### Discourse 1: Body as machine

“Body as a machine” was the most common discourse and was evident in almost all participant responses. Most participants viewed their body in a machine-like way – as if something mechanical was “wrong” with their body and that this caused their LBP recurrence or persistence. Described causes included: joint, muscle and nerve injury/disease; posture and “alignment” issues; muscle control, length and strength issues; and inflammatory conditions. Participants often presented a distinct picture of what they believed was wrong. For example, participant 3 wrote:

“Degeneration of the integrity of my tendons and ligaments from faulty collagen due to Ehlers-Danlos Syndrome causing instability in my spine (and other joints) resulting in herniation of spinal discs (currently 3 cervical, 1 thoracic and 2 lumbar) and degenerative disc disease at L5/S1. Also sacroiliac joint dysfunction, hip dysplasia and instability has a correlating impact to my back issues.”

And participant 59 wrote:

“My motor control has suffered due to chronic low back pain initially caused by an injury and then perpetuated by degeneration in the joints. Even though there is no acute injury any more (arthritis is still there), my motor patterns are inefficient and I recruit larger muscles to stabilise my back due to pain inhibition. This means sometimes I do movements that are actually more forceful that needed and increase joint loading at the degenerating level, which is what causes a flare up.”

Like these participants, many used technical biomedical language, for example: “fusion surgery leading to sacroiliac joint problems” P8, “my L4 and L5 are rubbing together” P43, and “spondylolisthesis L5S1 with pars defect” P62. Others spoke less specifically of general physical conditions (e.g., “spinal damage caused by arthritis” P7).

#### Discourse 2: LBP as permanent/immutable

“I am suffering from multi-level degenerating disks. (L1-S1) There is no "mechanical fix" for my condition. And as time goes by it continues to degenerate.” P105

Like the opening quote above, many responses included some indication that when LBP damage or disease occurred it would be that way forever. This discourse is related to the first, where participants viewed the body as machine-like (relatively immutable). For example, several participants referred to earlier damage that had a seemingly permanent or definitive ongoing effect: “Damage done earlier in life” P107, and “Injury from high school...” P85. These participants’ attribution of an ongoing cause of their condition seemed to indicate that a prior cause for their LBP meant that it could not be changed.

Akin to the opening quote, the word ‘degeneration’ was commonly used by participants to indicate that damage was ongoing and worsening. For example “Now, it has become a matter of degeneration to the structure due to age and injury” P49. Other participants referred to more specific diagnoses, in this way denoting that their condition was permanent. For example, one participant said “arthritic changes in the bones” P12, and another “severe multi-level stenosis” P97. One participant (59) stated clearly that, due to their diagnosis they believed their LBP is likely to persist and worsen, “My understanding is that because of my scoliosis I may always have lower back pain – and this could increase as I get older.” Overall, participants referred to damage, degeneration or LBP diagnoses as reified conditions, and almost never framed their LBP as something that is transient, reversible or temporary.

#### Discourse 3: LBP is complex

This discourse was much less common and contrasted with the first two discourses – it is a counter narrative to them. These participants indicated, at least in some way, that LBP can be complex; that bodies are not simply machines that are broken or not, but rather that bodies, disease states, and pain can be impermanent and complex – and variously reworked. Factors other than biomechanics and disease processes can contribute to LBP’s recurrence or persistence. For example: one participant said that her LBP’s persistence was “…in part my dependence on medication” P52, and another that it was “Pain patterns in brain as well as muscles that engage to 'protect' me when they don't need to.” P83. Participant 50 described considerable detail complex mechanical and psychological contributors:

“I have a severe burst dispersion fracture of L1 with up to 75% of the body of L1 crushed and dissolved. I have no neurological impairment and the fracture was stabilised without surgery. In 2013 I had a 20-year MRI and consulted a private pain specialist (also ortho surgeon) and he confirmed that the root cause is mechanical. My background pain was very high for approx 1 year (mid 2012-13) during a suicidal depression period. I have several month long bouts of depression every 3-5 years but the 2012 episode was worse than others. This fed the pain which fed the depression and I started hating my pain for the first time in 22 years. Although it can be tiring and exasperating at times, I had never hated the pain or wished it gone. Interestingly, during a few months of intense psychological treatment sessions, I had a week and a half long bad pain episode but it wasn't until the 4th day that I realised that my attitude to the pain and my "automatic responses" to it had reverted back to my usual acceptance so I saw that as a step forward. The year highlighted again the direct correlation of mood to pain.”

This participant highlights a complex interplay between biological, psychological and emotional contributing factors to his condition: while LBP may have an anatomical driver of a fractured vertebra, he believed his LBP was also impacted by the interplay of psychological health (depression), mood (anger) and pain beliefs (acceptance).

Participants who said they were unsure about what caused their condition also had a discourse of LBP as complex. For example, participants used statements such as: “I am not sure why this happens….” P43, “There is no understanding it, it is a combination so specific to the individual” P13, and another: “I have degeneration in the L4 and L5. No apparent reason why. Every physiotherapist, specialist, massage therapist and osteopath all have different theories why. Nothing conclusive as I have not had a significant trauma and the degeneration indicates that I have.” P69. The complexity and the lack of knowledge about LBP is portrayed as a mystery, and these participants give little indication of whether this enabled or disempowered them to manage their condition. Interestingly, however, one participant indicated that not being sure of the cause of their ongoing LBP did not stop them from taking action:

“I don't really know [why my pain is ongoing or recurrent]. I do believe that there's a "learned behaviour" in my brain. I have been trying to focus on different things lately or visualise different things when my back starts to get worse, and it seems to be helping a bit. I have recently gone back to the gym and have found that I can move and perform some exercises that not only are pain free but help with my overall pain. That has shown me that movement is not the cause of my pain, but the type of movement and how I do it.” P71

Two participants (P71 and P13 – both quoted above) *only* stated a complex picture of their condition without any aspect of the first two discourses (‘body as machine’ or ‘body as permanent’).

#### Discourse 4: LBP is very negative

Many people described their LBP very negatively, often explicitly stating this. For example, participant 3 said “Severe spinal stenosis and an awful scoliosis”, and participant 31 “severe sciatica...pain never goes away”. Participants frequently used negative words such as “damage”, “degeneration” to denote that they believed that their body was harmed. For example, participant 33 said: “I have worn out, my L5/S1 to the point, it can’t take anything else.” Often participants used the word “poor” (poor posture, poor disc health, poor core strength).

The discourse of LBP being very negative was also often implicit in discourse 1 and 2 (but was not classified this way for the sake of clarity in this analysis). In itself, the idea of the body as a machine which breaks permanently is a negative conceptualization of their condition. For example, return to the quote: “My understanding is that because of my scoliosis I may always have lower back pain – and this could increase as I get older.” P59. Although there are no overtly negative words used, the words ‘always’ and ‘increase’ give the statement a negative valence.

Although some participants were only mildly negative it was rare that people’s reasons given for the persistence or recurrence of their LBP were neutral or normalised, and extremely rare that they indicated a positive relationship to their LBP when thinking about its persistence or recurrence. One of the few examples of a more neutral, normalised and somewhat positive response was embedded within participant 50’s reply which was quoted in full above: “Although it can be tiring and exasperating at times, I had never hated the pain or wished it gone.”

### Analysis of responses to question 2

The second question was: “Where does this understanding come from?”. Findings are summarised in Table 3. Participants could choose one or more options - with most choosing only 1. Of the four possible options, the majority indicated that their understanding of their LBP came from a health professional (*n* = 116, 89%) and almost one quarter from the internet (*n* = 31, 24%), with the other options being chosen much less frequently. The option ‘other’ was selected by 49 people – however, some (*n* = 15) responses were simply clarifications of the other categories (so were excluded from consideration under ‘other’), one response was not relevant as it was unrelated to the question. The most pertinent findings were that a small number (*n* = 16, 12%) of people discussed reflection on their own experiences (e.g., “knowing my own body” P46 and “my own reflection” P63) as informing their understanding of why their LBP was ongoing. A small number also discussed information gathering from formal health education (*n* = 9) or scientific literature (*n* = 4). This study was not intended to detect statistically significant relationships between the discourses and the reported source of their understanding of causes. However, in a simple comparison there were no indications of potential relationships between the participants’ belief of where their LBP had come from and the discourses in their responses to question 1.

**Table 3:** Number (percentage) of responses to the question “Where does this understanding come from?”

Health Care Provider n (%)	Internet n (%)	Family n (%)	Friends n (%)	Other* n (%)
116 (89)	31 (24)	12 (9)	7 (5)	16 (12) self-reflection 9 (7) education 4 (3) scientific lit 3 (2) other 1 (1) not relevant
				**33 (25) Total**

*In the “other” box 15 participants provided clarification or repetition of the first four options. These figures were not included separately in the analysis

## Discussion

The key finding of this study is that people with LBP predominantly consider their condition to persist or recur because of biomechanical or structural reasons (machines that can be broken, and if not ‘fixed’ will continue to be in pain/damaged). Participants discussed factors such as joint damage, nerve injury and/or muscle imbalance as the main reasons why their LBP was ongoing. While there were counter-narratives, these were much less common. This finding indicates that overwhelmingly individual’s beliefs about their LBP are aligned with (Western) traditional biomedical discourses of health and the body. A secondary finding of this study was that participants overwhelmingly considered health professionals, and the internet to be sources of their understandings. This highlights the likely value of ensuring good quality information from both sources. This study can only discuss where people with LBP *say* they learnt about the causes of their condition and thus findings may not reflect what health professionals believe or do in their healthcare practice, nor the quality of internet resources. However, as discussed, other studies have shown that health professionals, such as physicians [26] and physiotherapists [27], demonstrate primarily biomedical pain beliefs and practices, and that this is strongly associated with the beliefs of their patients [36]. This primarily Western belief system has also been shown to have potential effects across cultures [37].

Why do the LBP-related discourses found in this study matter? There is considerable evidence to suggest harm is done by this way of thinking. People might modify their behaviour in a manner that may worsen their LBP, placing emphasis on avoiding causes that are not relevant. Greater beliefs in the anatomical causes of persistent pain have been related to greater beliefs in physical disability [17] and thus avoidance of activities. There are strong associations between low perception of controllability of LBP and poor clinical outcomes [19] and this belief is likely to underpin strongly negative beliefs about pain. Encouragingly, some people have a more complex understanding of LBP, aligned with more contemporary biopsychosocial discourses, but this was rare in our data and is often largely overshadowed by biomedical discourses.

Similar findings about beliefs of individuals with LBP have been reported elsewhere. For example, Bunzli, Watkins, Smith, Schütze and O’Sullivan [38] synthesized 18 studies with 713 participants with chronic LBP, highlighting the social construction of the condition, and its psychosocial impact. The study discussed that people with chronic LBP often adhere to a biomedical model of their condition and that this results in them “putting their lives on hold” until they receive what they believe to be a viable (biomedical) diagnosis/prognosis. Consistent with our findings, the synthesis suggests that the potential harm in these beliefs is that people may undertake a misdirected search for legitimacy that prevents both acceptance of the condition as well as attention to more evidence based contributors to continuing LBP. The present findings strengthen these observations using a novel approach to uncover underlying discourses and contribute to new understandings of the trajectories of LBP. The present study also reveals that biomedical discourses are prevalent despite many efforts towards changing these beliefs (e.g., [39]), and potential positive clinical outcomes of changing them (e.g., [40]).

Interpretation of these findings poses challenges. It is important to acknowledge we must take seriously the perspectives of people living with LBP [41]. Not doing so has led to issues in the past, such as inadvertent stigmatisation [42] and inadequate attention to the often-complex psychosocial aspects of living with ongoing conditions [43, 44]. If individual perspectives are predominantly produced by an outdated (or at least, incomplete) healthcare paradigm, it is necessary to consider how this might be challenged.

Our findings offer some possible ways forward, evident in that some participants’ responses included all discourses. This indicates that individuals with LBP are able to think with more than one paradigm – switching between them seemingly with little issue concerning apparent contradictions. For example:

“there is degeneration of vertebra and discs which results in pinching of nerves. A lot of soft tissue damage to core muscles front and back which makes that back more pliable. It is said that my brain is not interpreting the signals properly because of many things including PTSD, TBI, IBS…” P2.

This participant used all four discourses – but some are stronger than others. The participant speaks first about biomechanical/anatomical factors as reasons for the ongoing nature of their LBP (“degenerated vertebra and discs”, “pinching of nerves”, “soft tissue damage”) using very definite language “there is…results in… a lot of…”. This language suits the dominant form of understanding bodies and health in biomedicine: bodies are like machines that may or may not be fixed. Like a machine, when something cannot be ‘fixed’ it is ‘permanently broken’. Notably, the participant also provides another less definite story (“it is said that...”). This choice of wording makes the subsequent statement appear not to be coming from her/his own thinking, perhaps indicating the participant has heard this information but does not really believe/understand it. In this second story, the participant discusses that LBP can persist because a number of factors can influence it (the participant mentions psychological and other physical conditions). Although the traditional view is given more emphasis, the presence of both stories suggests this person is able to adopt more than one perspective – an understanding of LBP as complex and not only biomechanical or anatomical. Such participants show an ability to integrate biopsychosocial with biomedical understandings. As other research has highlighted that sense-making processes may play a role in developing harmful LBP beliefs [45], this highlights a potentially useful way forward to assist people with LBP (and others) to helpfully reconceptualise their condition.

The findings of the discourse ‘LBP as very negative’ were often closely linked with the understanding of the body as ‘a machine that can break’ and as ‘permanent’. This close relationship highlights further that underlying discourses have important effects on beliefs about LBP, which in turn affect prognosis. The conceptualisation of LBP as very negative found here and elsewhere [21] is not surprising given the underlying discourses of the body as a machine that can break, and LBP as permanent. Yet this conceptualistation contrasts with the fact that LBP is so common as to say that it is normal [2], and persistence/recurrence are also very common/normal [4]. Although not directly researched regarding LBP itself, other conditions involving injury, illness and disability have been conceptualised differently including positive possibilities such as those of ‘well-being within illness’ [46], hope [47], and acceptance [48]. These possibilities for more positive reconceptulisations might be a relevant to consider for future work in the LBP population. If LBP was perceived as relatively normal (although at times unpleasant) rather than something to always ‘fix’, potentially harmful beliefs and their negative psychosocial and clinical implications might be avoided.

There are a number of important factors to consider when interpreting the results of this study. Although survey formats have some limitations in qualitative research due to the lack of direct interaction, there are also benefits - due to the online nature of this study, participants were likely to have felt comfortable expressing their views freely. The study was Australian, consequently, care must be taken in extrapolating results to other settings – although similar Western countries are likely to find many contextual similarities. The investigatory team was comprised of researchers with a physiotherapy background (and one social worker). Physiotherapists would be expected to have views on LBP grounded in biomedical aspects and may give less attention to psychological and socio-cultural aspects [49]. Efforts were made to reduce any effect of this viewpoint by assembling a team that includes a social worker (MN), a researcher with graduate training in psychology who focuses on the socio-cultural elements of health (JS), and sourcing external review. Future research can build on the findings of this study. A similar research design could be conducted in different cultural contexts to investigate how LBP is conceptualised elsewhere.

Finding that biomedical discourses underlie most thinking about LBP has implications for how it is possible to change. These discourses challenge deep cultural understandings of what it means to have a body and to be human, including the Cartesian notion of a distinct body/mind division [50]. Changing such deeply held beliefs will not be simple – doing so challenges established institutions of healthcare and the very core of what it means to be a person with LBP, a health researcher, and a clinician [51]. This study provides a strong basis for challenging deeply held traditional beliefs and interventional studies testing these as methods for reducing the burden of LBP are indicated.

We do not want to deny possible biomedical causes of LBP or permanence of the condition. Rather, our aim is to highlight that these discourses are not necessarily true for everyone and can set people up for damaging pain beliefs such as fear avoidance. Described biomedical causes in the current study included various musculoskeletal injuries/diseases and biomechanical factors. Although some biomedical factors have support as the initial cause of LBP [13], there is currently little support for them causing ongoing or recurrent LBP which, as discussed, is likely to be more biopsychosocial and multifactorial [8–11]. There is thus a disconnection between cause of initial injury and the reason it is ongoing, which is worthy of further investigation.

## Conclusions

The findings of this study support and add to other studies discussing that biomedical understandings of LBP persist and recur in those who live with musculoskeletal pain conditions. Seeking to understand underlying discourses provides a novel perspective which looks past singular causes to consider the systems of thought that make an adherence to particular patterns of beliefs possible. The findings of this study support a complex and thorough approach to shifting understandings of LBP beyond biological causes to consider psychosocial, cultural and institutional factors that constitute LBP. Finally, our finding that patients believe they learnt their potentially harmful understandings from health professionals encourages further interventions to shift thinking within healthcare.

## Abbreviation

LBP: Low back pain
